# Anxiolytic Effects of Indole Alkaloids From *Rauvolfia ligustrina* in Adult Zebrafish: Involvement of the GABAergic and 5‐HT Systems

**DOI:** 10.1002/cbdv.202500016

**Published:** 2025-06-03

**Authors:** Nádia Aguiar Portela Pinheiro, Ivana Carneiro Romão, Amanda Maria Barros Alves, Sônia Maria Costa Siqueira, Jane Eire Silva Alencar de Menezes, Emmanuel Silva Marinho, Márcia Machado Marinho, Herbert de Sousa Magalhães, Andreia Ferreira de Castro Gomes, Otília Deusdênia Loiola Pessoa, Hélcio Silva dos Santos

**Affiliations:** ^1^ Graduate Program in Natural Sciences State University of Ceará Fortaleza Brazil; ^2^ Center for Exact Sciences and Technology State University of Vale do Acaraú Sobral Brazil; ^3^ Department of Organic and Inorganic Chemistry Federal University of Ceará Fortaleza Brazil; ^4^ Department of Biology, Environmental Biology, School of Sciences University of Minho Braga Portugal

**Keywords:** anxiety, DMPK, indole alkaloids, molecular docking, zebrafish

## Abstract

Benzodiazepines are widely used in the treatment of anxiety, but their use may be interrupted due to side effects. This study evaluated the indole alkaloids isocarapanaubine (indole alkaloid 1 [AIN1]) and 18‐β‐hydroxy‐3‐epi‐α‐yohimbine (indole alkaloid 2 [AIN2]), extracted from *Rauvolfia ligustrina*, in adult zebrafish. Three doses (4, 12, and 20 mg/kg) were tested, along with dimethyl sulfoxide (DMSO) 3% and Diazepam (4 mg/kg) in toxicity, locomotion, and anxiolytic effect assays. AIN1 and AIN2 showed low toxicity (lethal dose capable of killing 50% of the animals >20 mg/kg) and reduced locomotor activity. Both exhibited anxiolytic effects, with AIN1 acting through the GABAergic system and AIN2 via the serotonergic pathway (5‐HT_3A_). Interactions with receptors were confirmed by molecular docking, and drug metabolism and pharmacokinetics studies indicated that AIN2 is promising in terms of permeability and safety in the central nervous system.

## Graphical abstract

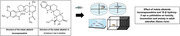

## 1 Introduction

The World Health Organization reports that one in eight people suffers from mental disorders, with a significant increase following the coronavirus disease 2019 pandemic [1]. More than 1 billion people worldwide suffer from mental, neurological, and substance use disorders [2]. Treatment often includes the use of anxiolytics such as benzodiazepines, selective serotonin reuptake inhibitors, β‐adrenergic antagonists, and azapirones [3]. However, unwanted side effects may occur, such as sexual problems, cardiovascular toxicity, drug interactions, and weight gain as a result of treatment, which may lead to discontinuation of treatment [4]. Thus, treatment for anxiety disorders requires ongoing research focused on investigating new compounds that offer better therapeutic profiles and reduce side effects [5].

In this context, alkaloids emerge as a promising research alternative. They are present in various plant species and are a chemical characteristic of the *Apocynaceae* family [6]. In the *Rauvolfia* genus, the presence of indole alkaloids has been studied for a long time [7]. These alkaloids have a bicyclic structure, with a benzene ring attached to a pyrrole ring, similar to the structure of serotonin, a neurotransmitter essential for mood balance and brain functions [8]. Several indole alkaloids have been described in the literature with promising results in the treatment of anxiety, such as sarpagine, 3‐hydroxysarpagine, 17‐epi‐rauvovertine B, Peraksine, 17‐epi‐peraksine B^9^, Alstonine [10], 9‐methoxy‐2‐phenyl‐11H‐indolisino[8,7‐beta]indole [11], and Psicolatine [12], all showing positive results, except for 3‐hydroxysarpagine. However, studies on the indole alkaloids Isocarapanaubine and 18‐β‐hydroxy‐3‐epi‐α‐yohimbine have primarily focused on their phytochemical study and isolation, with a significant gap remaining in the investigation of their anxiolytic properties.

To study the causes and effects of human diseases, science relies on animal models. Thus, zebrafish, a small freshwater fish with genomes similar to humans, is excellent for investigating various diseases and allowing testing for new treatments [13, 14].

**FIGURE 1:**
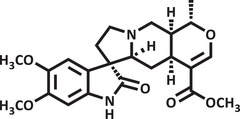
Structural representation of isocarapanaubine.

**FIGURE 2:**
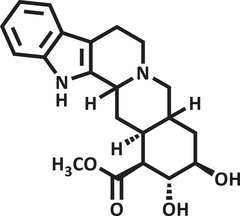
Structural representation of 18‐β‐hydroxy‐3‐epi‐α‐yohimbine.

Therefore, due to the biological potential of indole alkaloids for the development of new drugs, combined with the increasing cases of anxiety in society, the value of searching for new substances that act on the central nervous system is clear. Thus, using zebrafish as an animal model and conducting a pharmacodynamic analysis (molecular docking) and pharmacokinetic analysis (drug metabolism and pharmacokinetics [DMPK]) of the substances, the objective of this study was to evaluate the toxicity and anxiolytic activity of the indole alkaloids isocarapanaubine (AIN1) and 18‐β‐hydroxy‐3‐epi‐α‐yohimbine (AIN2), obtained from *Rauvolfia ligustrina*, as well as to assess their mechanism of action (Figures 1 and 2).

## 2 Results and Discussion

### 2.1 Acute in Vivo Toxicity

Zebrafish is an animal known for its susceptibility to intoxication by toxic agents [15]. As such, this model has been widely used since the development of new drugs depends on toxicological assessment to anticipate potential risks, thereby reducing the chance of harming human health [16]. Therefore, samples AIN1 and AIN2 showed no toxicity over 96 h at the studied doses (4, 12, and 20 mg/kg). These data allowed for the continuation of the study with the in vivo open‐field behavioral test, aiming to identify safe doses with possible activities in the central nervous system of zebrafish.

The toxicity of indole alkaloids has already been reported in the literature, such as in the research by Sousa et al. [9], who studied sarpagine, 3‐hydroxy‐sarpagine, sarpaginine, 17‐epi‐peraksisna, peraksine, 17‐epi‐rauvertina B, and rauvovertina B, indole alkaloids isolated from *R. ligustrina*, at doses of 4, 20 and 40 mg/kg, with none showing toxicity to zebrafish, reinforcing the data described in the present study.

**FIGURE 3:**
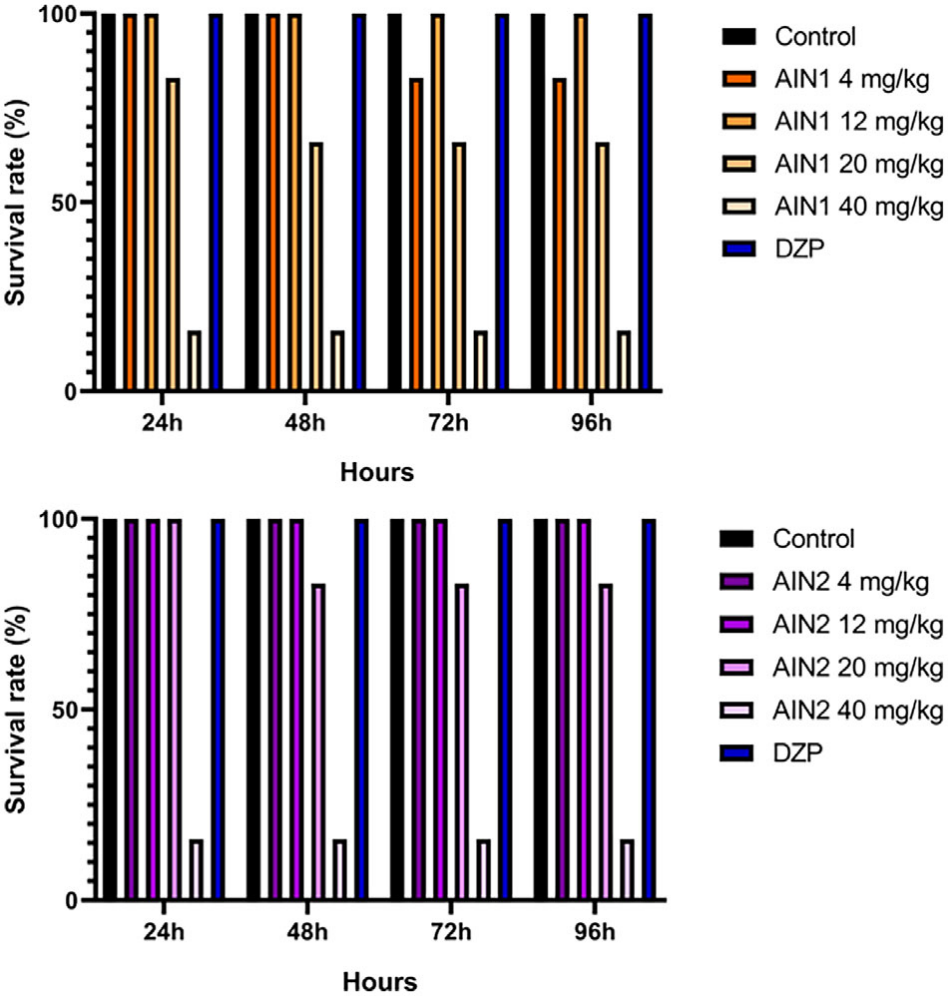
Effect of indole alkaloid dosages on zebrafish survival rate. DZP—diazepam (4 mg/kg; 20 µL/animal; i.p.). Control—DMSO (3%; 20 µL/animal; i.p.).

The graphs in Figure 3 showed that the AIN1 and AIN2 samples, at the doses selected for the study, did not exhibit toxicity, as the fish survival rate was above 50%, which is the threshold considered in lethal dose capable of killing 50% of the animals (LD_50_) analysis. However, the 40 mg/kg dose of both samples proved to be toxic to the fish, showing a survival rate of approximately 15%.

### 2.2 Evaluation of Locomotor Activity

**FIGURE 4:**
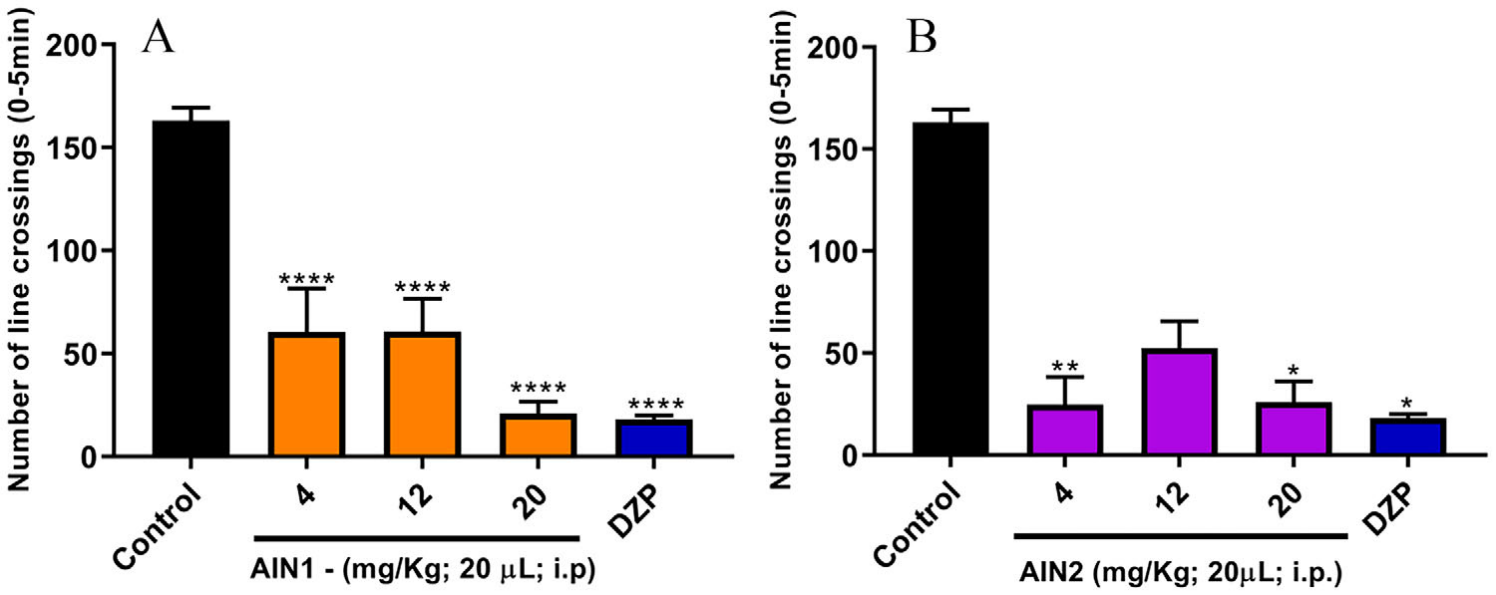
Effect of indole alkaloids on the locomotor activity of zebrafish. DZP—diazepam (4 mg/kg; 20 µL/animal; i.p.). Control—DMSO (3%; 20 µL; i.p.). The values represent the mean ± standard error of the mean (S.E.M.) for 6 animals/group. Analysis of variance (ANOVA) followed by Tukey for the isocarapanaubine (AIN1) sample and Kruskal‐Wallis test for the 18‐β‐hydroxy‐3‐epi‐α‐yohimbine (AIN2) sample; *p < 0.05; **p < 0.01, ****p < 0.0001 versus Negative control.

The open field test is one of the gold standard assays in preclinical studies and is commonly used to investigate compounds with activity in the nervous system, as well as to confirm their efficacy [17]. In this test, the locomotor activity of the animals was significantly altered when compared to the negative control (LC = 163 ± 6.38) in the line crossing (LC) analysis, at least at one dose of each sample studied (Figure 3). AIN1 showed this difference at all tested doses (****p < 0.0001) with LC = 60.50 ± 21.09; LC = 60.67 ± 15.85; LC = 20.83 ± 5.91 for doses of 4, 12, and 20 mg/kg, respectively (Figure 4A); while AIN2 was distinguishable from the negative control at doses of 4 mg/kg (**p < 0.01) with LC = 24.67 ± 13.74 and 20 mg/kg (*p < 0.05) with LC = 25.83 ± 10.40 (Figure 3B). It is noteworthy that the positive control showed a result equal to LC = 18 ± 2.02.

The absence of alteration in the locomotor activity of the AIN2 sample at the intermediate dose (12 mg/kg) (Figure 4B) can be attributed to the phenomenon known as the inverted U‐shaped dose‐response curve or biphasic response. This type of response is characterized by significant biological effects occurring only at low and high doses of a substance, while intermediate doses do not produce substantial changes. In the context of this study, it is particularly relevant that the sample did not cause changes in the fish locomotor system but still demonstrated anxiolytic effects. This suggests that the sample has the potential to act as an anxiolytic agent without inducing the sedative effects observed in the positive control (diazepam [DZP]), indicating a promising pharmacological profile for anxiety treatment [18].

Corroborating the data from the present study, Sousa et al. [9] found alterations in the locomotion of zebrafish for all the studied indole alkaloids at least at one of the evaluated doses (4, 20, and 40 mg/kg) when compared to the negative control. Additionally, the research by Sakakibara et al. [19] investigated the effect of indole alkaloids corinoxine (30 mg/kg), corinoxine B (30 mg/kg), isorincofiline (100 mg/kg), and methyl ether of geissoschizine (100 mg/kg) on the locomotor activity of mice and concluded that there was a significant decrease.

### 2.3 Evaluation of Anxiolytic Activity

**FIGURE 5:**
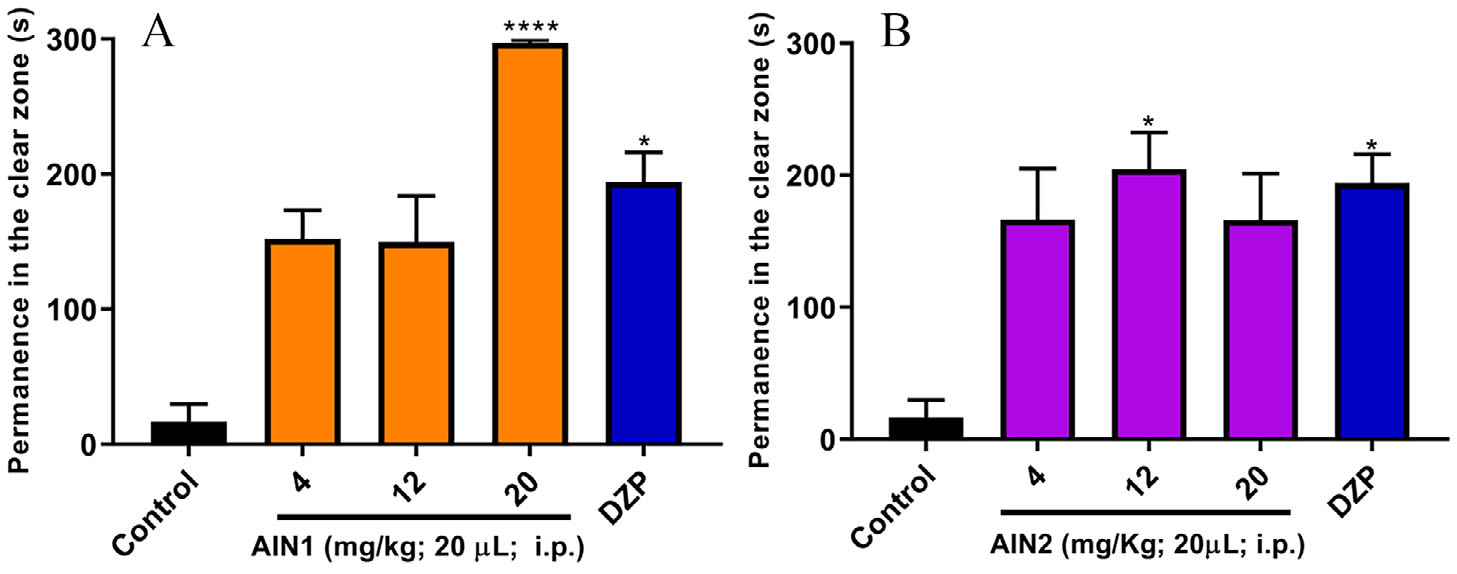
Anxiolytic effect of indole alkaloids in adult zebrafish in the light/dark test. DZP—diazepam (4 mg/kg; 20 µL/animal; i.p.); Negative control –DMSO (3%; 20 µL; i.p.). The values represent the mean ± standard error of the mean for 6 animals/group; Kruskal‐Wallis test; *p < 0.05; ****p < 0.0001 versus Negative control.

The light/dark test evaluates the animal's behavior in response to anxiety and fear. Samples with anxiolytic characteristics allow animals to spend more time in the illuminated area of the aquarium while anxiety‐inducing drugs reduce this time. The anxiolytic activity was assessed using the light/dark test by measuring the time spent in the light zone (TSLZ) of the aquarium, and the samples exhibited an anxiolytic effect in adult zebrafish when compared to the negative control (TSLZ = 16.67 ± 13.10), as shown in Figure 5. The alkaloid AIN1 showed a TSLZ = 297.17 ± 2.29 for the 20 mg/kg dose (Figure 5A), and the alkaloid AIN2 showed a TSLZ = 204.50 ± 27.85 for the 12 mg/kg dose (Figure 5B), similar to the DZP‐treated group (TSLZ = 194.00 ± 22.18). As a result, the best doses for AIN1 and AIN2 were 20 mg/kg and 12 mg/kg, respectively, as they exhibited anxiolytic activity without significant adverse effects. It is worth noting that AIN1 demonstrated a visually superior anxiolytic effect compared to DZP at the 20 mg/kg dose (Figure 5A).

### 2.4 Evaluation of GABAergic and Serotonergic Neuromodulation

**FIGURE 6:**
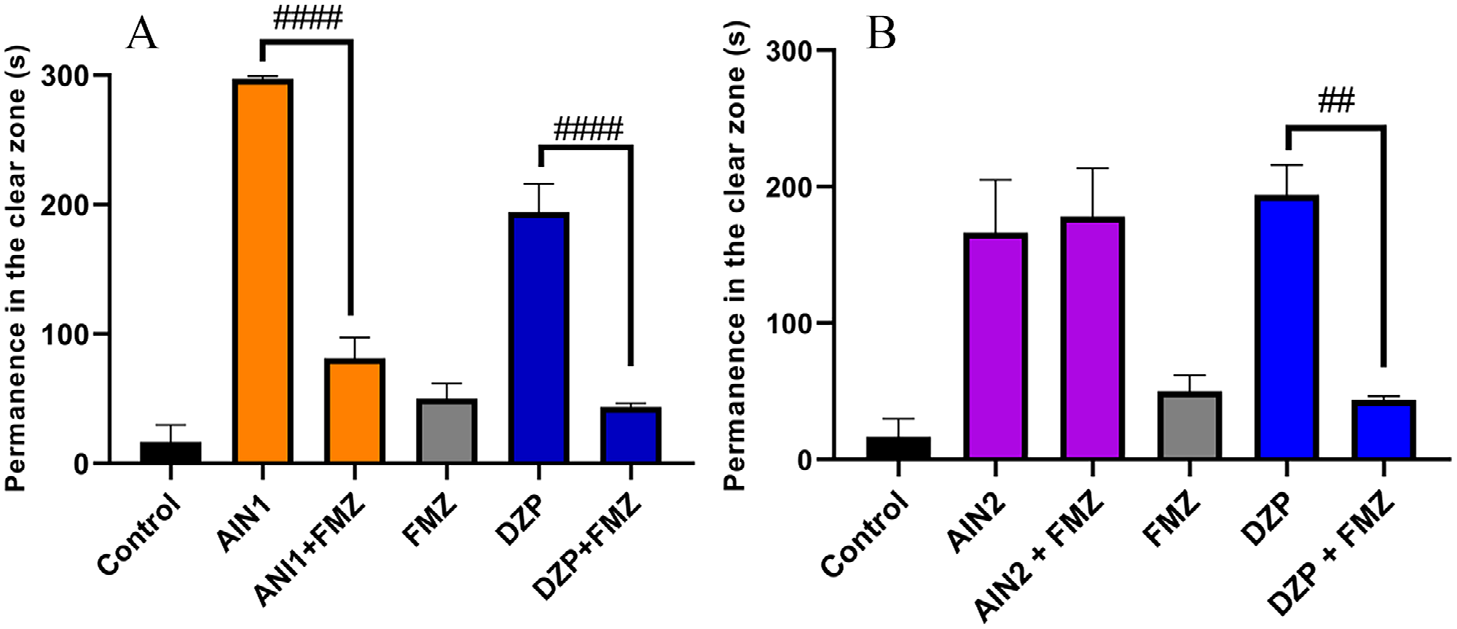
(A) Evaluation of isocarapanaubine (AIN1) and its interaction with flumazenil (FMZ) and diazepam (DZP). ####*p* < 0.0001 AIN1 versus AIN1 + FMZ; ####*p* < 0.0001 DZP versus DZP + FMZ. (B) Evaluation of 18‐β‐hydroxy‐3‐epi‐α‐yohimbine (AIN2) and its interaction with FMZ and DZP. ##*p* < 0.01 DZP versus DZP + FMZ. DZP (4 mg/kg; 20 µL/animal; i.p.); FMZ (4 mg/kg; 10 µL i.p.); Negative control –DMSO (3%; 20 µL/animal; i.p.). The values represent the mean ± standard error of the mean for 6 animals/group; Two‐way analysis of variance (ANOVA) followed by Tukey.

The anxiolytic mechanism of action was investigated via GABAergic (GABA_A_) and 5‐HT using the best doses with anxiolytic effects of the indole alkaloids AIN1 (20 mg/kg) and AIN2 (12 mg/kg). The alkaloid AIN1 had its anxiolytic effect blocked by FMZ (TSLZ = 81.17 ± 16.30) (####*p* < 0.0001 AIN1 vs. AIN1 + FMZ), indicating that the sample acted via GABA_A_ (Figure 6A), similar to what occurred with DZP (TSLZ = 43.50 ± 3.17) (####*p* < 0.0001 DZP vs. DZP + FMZ).

**FIGURE 7:**
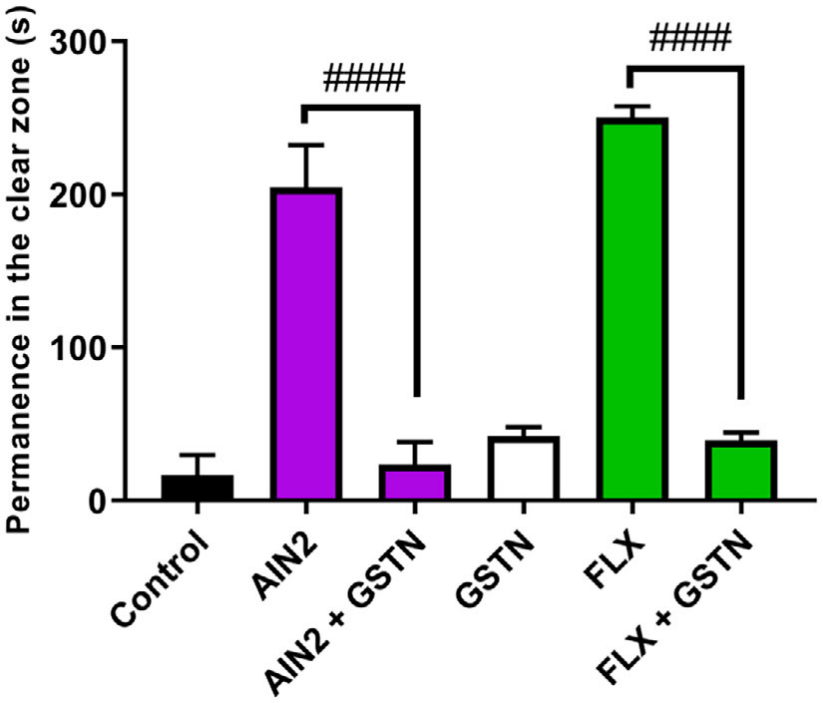
Anxiolytic action mechanism via 5‐HT3A of the 18‐β‐hydroxy‐3‐epi‐α‐yohimbine (AIN2) sample. GSTN—Granisetron (20 mg/kg; i.p.); FLX—Fluoxetine (0.05 mg/kg; oral); Negative control—DMSO (3%; 20 µL/animal; i.p.). The values represent the mean ± standard error of the mean for six animals/group; Two‐way analysis of variance (ANOVA) followed by Tukey. ####*p* < 0.0001 AIN2 versus AIN2 + GSTN; ####*p* < 0.0001 FLX versus GSTN + FLX.

However, the AIN2 alkaloid did not have its effect reversed via GABA_A_ (Figure 6B) and was subsequently investigated through 5‐HT_3A_, showing its effect to be significantly blocked by the granisetron (GSTN) antagonist (####*p* < 0.0001 AIN2 vs. AIN2 + GSTN) with TSLZ = 23.5 ± 14.88, similar to how fluoxetine (FLX) blocked it with TSLZ = 39.33 ± 5.38 (####*p* < 0.0001 FLX vs. FLX + GSTN) (Figure 7). As a result, the animals reverted to anxious behavior, spending more time in the dark zone of the aquarium.

The anxiolytic potential of indole alkaloids is well‐established in the literature. The study by Sousa et al. [9] showed that the indole alkaloids sarpagine, sarpaginine, 17‐epi‐peraksisna, peraksine, 17‐epi‐rauvertina B, and rauvovertina B exhibited similarities to DZP, indicating that they possess anxiolytic characteristics. The authors investigated the mechanism of action and concluded that all compounds acted via GABA_A_ pathways, except for sarpagine, which demonstrated action through the serotonergic system.

Additionally, Costa‐Campos et al. [10] used the light/dark test in mice to evaluate the anxiolytic activity of alstonine and obtained positive results with a serotonergic mechanism of action. Both et al. [12] studied the indole alkaloid psicotina at doses of 7.5 and 15 mg/kg, finding that it exhibited anxiolytic effects involving serotonergic receptors.

### 2.5 Molecular Docking Analysis

#### 2.5.1 GABA_A_R Interaction Analysis

**FIGURE 8:**
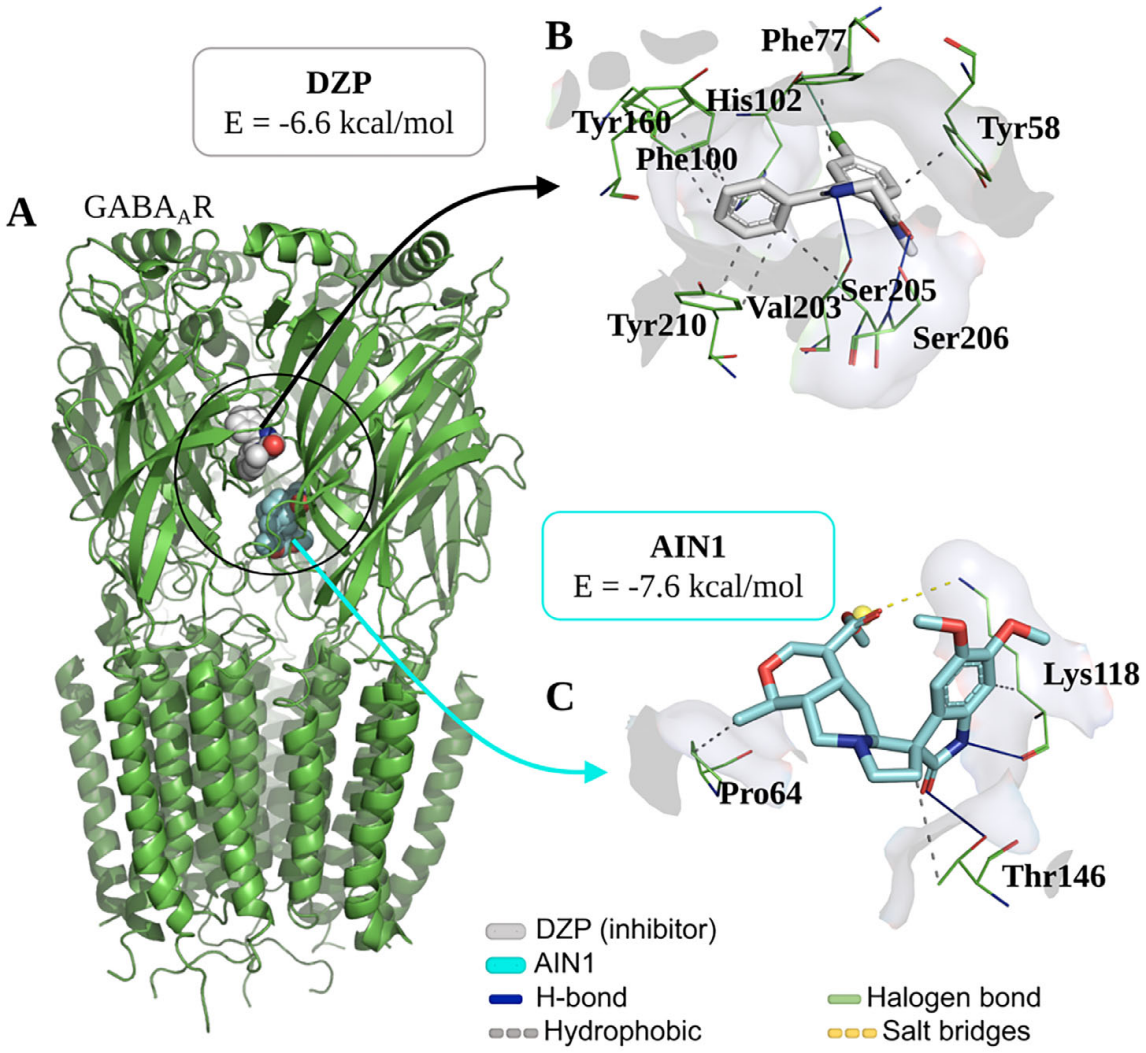
(A) Three‐dimensional representation of the isocarapanaubine (AIN1) ligand docking on the GABA_A_R receptor in relation to the diazepam (DZP) inhibitor and ligand‐receptor interactions of (B) DZP inhibitor (grey color) and (C) AIN1 (cyan color) in relation to amino acid residues (green color) of the GABA_A_R receptor.

At the end of the independent molecular docking simulation cycle via GABA_A_R, it was observed that the ligand AIN1 bound to the receptor at a distinct binding site from that of DZP (Figure 8A), although it interacts with residues in the C chain, where the DZP binding site is located, forming an AIN1/GABA_A_R complex with an affinity energy of approximately −7.6 kcal/mol (Table 1), which is within an ideal specificity threshold (E < −6.0 kcal/mol) [21].

**TABLE 1:** Data from molecular docking simulations expressed in statistical parameters (root mean square deviation [RMSD]), energetics, and details of ligand‐receptor interactions.

Ligand	Target	RMSD	Energy	Interactions			
				Type	Residue	Chain	Distance
GABA_A_R	AIN1	1.824 Å	‒7.6 kcal/mol	Hydrophobic	Pro64	C	3.97 Å
					Lys118	C	3.54 Å
					Thr146	C	3.95 Å
				H‐bond	Lys118	C	2.23 Å
					Thr146	C	3.35 Å
				Salt bridges	Lys118	C	4.39 Å
	Diazepam^*^	1.495 Å	‒6.6 kcal/mol	Hydrophobic	Tyr58	C	3.52 Å
					Phe77	C	3.47 Å
					Phe100	D	3.83 Å
					Phe100	D	3.92 Å
					Tyr160	D	3.56 Å
					Val203	D	3.95 Å
					Tyr210	D	3.38 Å
					Tyr210	D	3.86 Å
				H‐bond	Ser205	D	2.94 Å
					Ser206	D	3.16 Å
				Halogen bond	His102	D	3.82 Å
5‐HT_3A_R	AIN2	1.876 Å	‒8.9 kcal/mol	Hydrophobic	Trp63	B	3.41 Å
					Arg65	B	3.70 Å
					Phe199	C	3,69 Å
					Ile201	C	3.25 Å
					Tyr207	C	3.36 Å
				H‐bond	Asp42	B	2.26 Å
					Arg65	B	3.53 Å
				Salt bridges	Arg65	B	5.46 Å
	Fluoxetin^*^	1.637 Å	‒7.1 kcal/mol	Hydrophobic	Ile44	A	3.44 Å
					Ile44	A	3.70 Å
					Ile180	A	3.90 Å
					Tyr207	B	3.80 Å
				H‐bond	Asn101	B	3.28 Å

* Comparative ligand used in molecular docking simulations.

All simulations performed within an ideal statistical threshold assessed by root mean square deviation (RMSD) < 2.0 Å, indicating that the binding poses of the ligands exhibited a low root mean square deviation, allowing for the reproducibility of this simulation protocol (Table 1).

When analyzing the ligand‐receptor interactions, it was observed that DZP forms a series of hydrophobic interactions through its aromatic systems, particularly with the aromatic portion of the residues Tyr58, Phe77, Phe100, Tyr160, and Tyr210 (Figure 8B), constituting the key residues for GABA_A_R receptor modulation [22]. On the other hand, it is noteworthy that AIN1 formed H‐bond interactions with the receptor through its heterocyclic amide system (Figure 8C), where the nitrogen group interacted with the H‐bond accepting portion of the residue Lys118 (NH···O ═ C), and the carbonyl portion acted as an H‐bond acceptor in relation to the residue Thr146 (C ═ O···H─O), with donor‐acceptor distances of 2.23 and 3.35 Å (Table 1), respectively, indicating moderate strength interactions [23]. This analysis suggests a possible synergistic effect of AIN1 associated with the presence of DZP, enhancing the anxiolytic effect.

#### 2.5.2 5‐HT_3A_R Interaction Analysis

**FIGURE 9:**
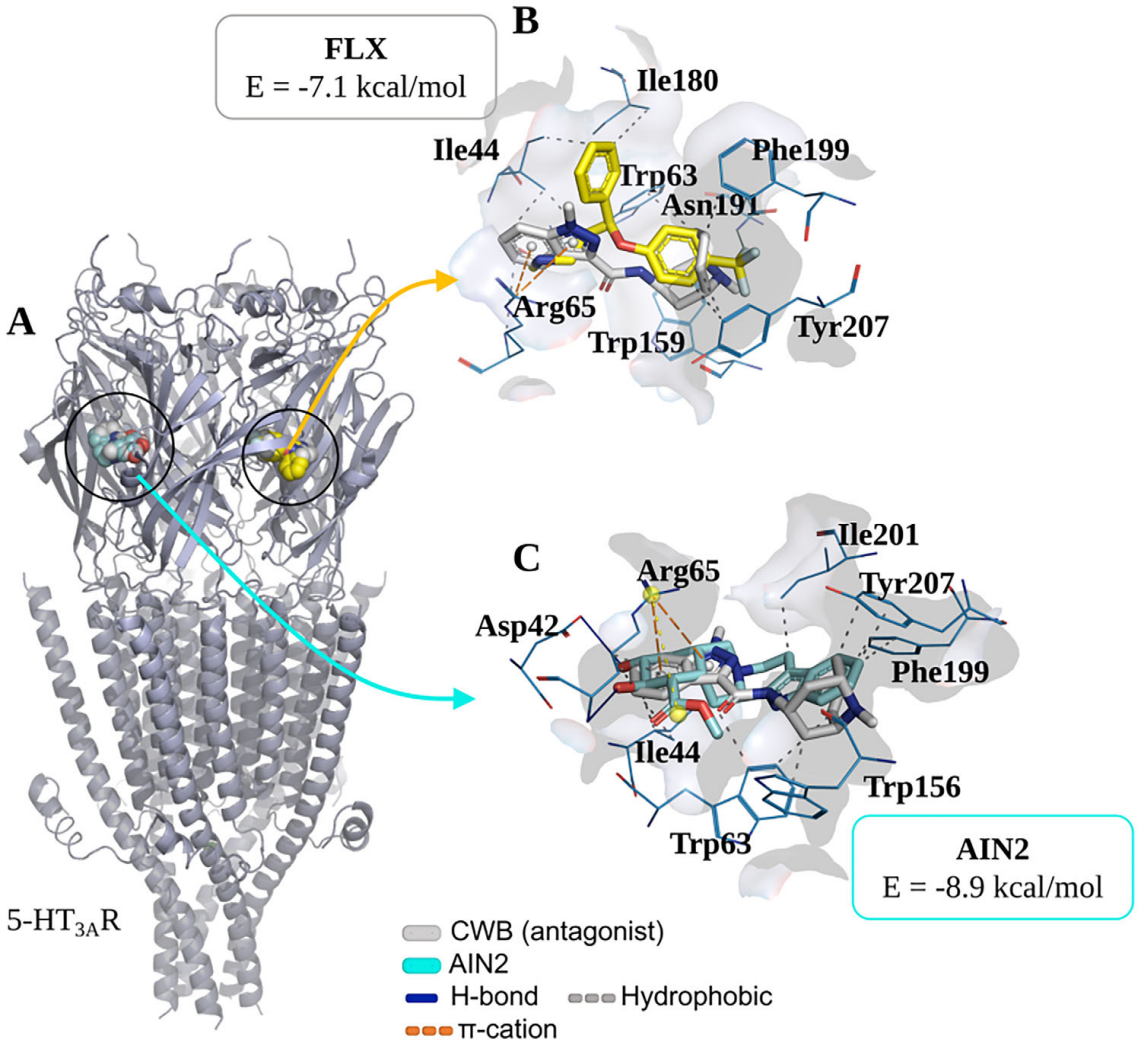
(A) Three‐dimensional representation of the docking of the 18‐β‐hydroxy‐3‐epi‐α‐yohimbine (AIN2) ligand on the 5‐HT_3A_R receptor in relation to the CWB antagonist and ligand‐receptor interactions of (B) FLX (yellow color) in relation to the CWB antagonist (grey color) and (C) of AIN2 (cyan color) in relation to the CWB antagonist (blue color) in relation to the amino acid residues (green color) of the 5‐HT_3A_R receptor.

In evaluating the serotonergic mechanism (5‐HT_3A_R), it was found that the derivative AIN2 can interact with residues at the binding site of the co‐crystallized inhibitor CWB (Figure 9A), located between chains B and C (Table 1). In contrast, the control FLX binds to the CWB binding site located between chains A and B (Figure 9A). At the end of the simulation cycles, an affinity energy of –8.9 kcal/mol was evaluated for the formation of the AIN2/5‐HT_3A_R complex, indicating a more energetically favorable coupling pattern compared to the drug FLX, which had a calculated value of –7.1 kcal/mol (Table 1) [21]. The simulations showed an RMSD < 2.0 Å, demonstrating a binding pattern with low root mean square deviation (Table 1).

When analyzing the ligand‐receptor interactions, it was observed that FLX interacts with residues at the CWB binding site between chains A and B, including a hydrophobic interaction with the aromatic portion of Tyr207 (B chain), through its aromatic ring with a trifluoromethyl radical (Figure 9B). Similarly, the ligand AIN2 presents a heterocyclic aromatic center capable of forming hydrophobic interactions with the residue Tyr207 (C chain), in addition to forming an H‐bond interaction with Arg65 (B chain), where the hydroxyl group acts as an H‐bond donor to the carbonyl group of the residue (O─H···O ═ C), showing that the ligand binds at the CWB binding site between chains B and C (Figure 9C). These analyses indicate that the compound AIN2 may act as an antagonist of the serotonergic mechanism.

Through molecular docking, Sousa et al. [9] analyzed several indole alkaloids, including sarpagine, 3‐hydroxy‐sarpagine, and ligustrine. The authors concluded that the sarpagine nuclei operated through the GABA_A_ system, with the interactions of 3‐hydroxy‐sarpagine occurring at the same site as DZP, while sarpagine acted at a distinct site, similar to AIN1 in the present study. Regarding ligustrine, it showed an affinity for the serotonergic receptor, acting at a different site than risperidone (the reference drug used), similar to AIN2 when compared with FLX. It is important to highlight that no molecular docking data were found for any of the samples under study.

### 2.6 In Silico DMPK Analysis

#### 2.6.1 Central Nervous System Multiparameter Optimization Analysis

A multiparameter optimization (MPO) model developed by Pfizer, Inc. is capable of estimating the pharmacokinetic profile of small molecules with suitable properties for the central nervous system (CNS). According to Wager et al. [24], weak bases with low lipophilicity (logP ≤ 3) that are larger and more polar (topological polar surface area [TPSA] 40–90 Å^2^) than commonly active compounds in the CNS demonstrate a positive correlation between safety and in vitro absorption, distribution, metabolism, and excretion (ADME) properties: high apparent permeability (Papp in 10⁻⁶ cm/s), low passive efflux mediated by P‐glycoprotein (P‐gp), and low intrinsic clearance in plasma (CLint,u < 8.0 mL/min/kg) [25]. These attributes are associated with oral absorption and metabolic stability of drugs.

**FIGURE 10:**
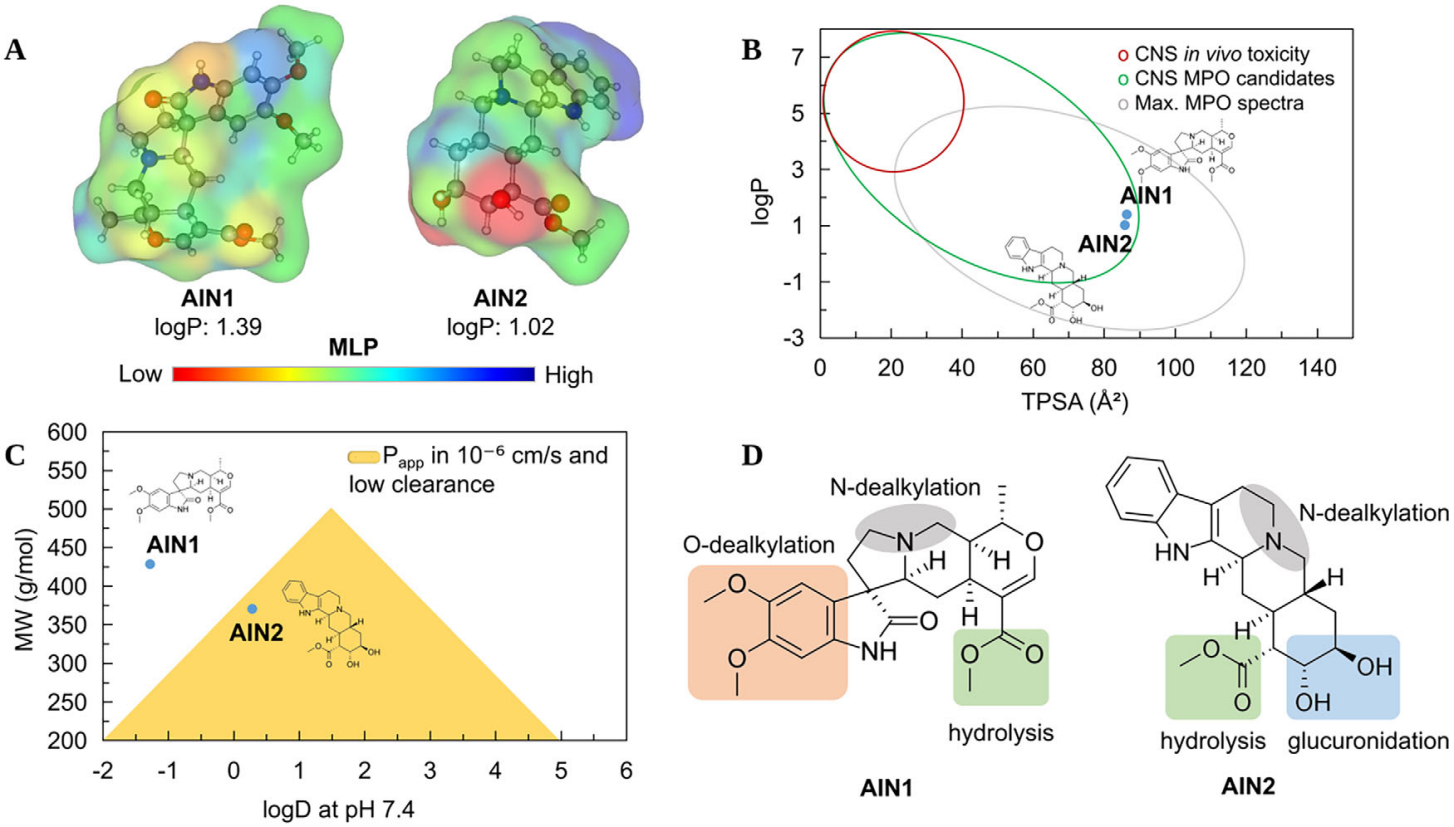
(A) surface map of molecular lipophilicity potential (MLP), (B) alignment between logP and topological polar surface area (TPSA) in the prediction of the permeability in the central nervous system (CNS), (C) alignment between solubility at physiological pH (logD) and molecular weight in estimating the balance between permeability and metabolic stability, and (D) site of metabolism prediction.

Topological analysis of the molecular lipophilicity potential (MLP) can reveal relationships between the lipophilic surface and the polar surface of small molecules, highlighting their permeability and diffusion potential across the cellular lipid bilayer [26]. In this analysis, it was observed that the derivative AIN1 exhibited a higher molecular weight (MW > 400 g/mol) and, by extension, a larger molecular surface compared to the derivative AIN2. However, it was noted that AIN2 has two hydrogen bond donor (HBD) groups of the OH type, resulting in a more hydrosoluble molecular surface (red color spectra) compared to AIN1, where the presence of polar hydrogen bond acceptor groups results in a less hydrophilic surface (yellow to blue spectra), as shown in the MLP map in Figure 10A, resulting in a logP of approximately 1.39 for AIN1 (most lipophilic) and 1.02 for the derivative AIN2 (less lipophilic).

The alignment of these descriptors resulted in a CNS MPO score of 4.26 for AIN1, due to its high MW, and 5.15 for AIN2, indicating a better active profile in the CNS for the AIN2 derivative (Table 2).

**TABLE 2:** Physicochemical properties of isocarapanaubine (AIN1) and 18‐β‐hydroxy‐3‐epi‐α‐yohimbine (AIN2) calculated and applied to the MPO score system.

Compound	logP	logD_7.4_	MW (g/mol)	TPSA (Å^2^)	HBD	pKa		CNS MPO	Pfizer rule
AIN1	1.39	−1.28	428.49	86.33	1	10.14	4.26		Optimized	
AIN2	1.02	0.28	370.45	85.79	3	8.05	5.15		Optimized	

When the lipophilicity (logP) and topological polarity (TPSA) descriptors were aligned, it was possible to observe that the compounds AIN1 and AIN2 reside in a physicochemical space that is safe for the CNS, especially due to logP values < 3 and TPSA > 75 Å^2^ (Figure 10B). Supporting this, the MDCK Papp descriptors were greater than 1.0 × 10⁻⁶ cm/s, favoring the passive permeability of AIN2 across the BBB (Figure 10C) (Table 3), indicating gradual access to the CNS [25]. On the other hand, when the solubility attributes at physiological pH (logD at pH 7.4) were aligned with MW, it was observed that AIN2 is in a favorable physicochemical space for passive intestinal permeability and low hepatic clearance, agreeing with the estimated Caco‐2 Papp of 1.3 × 10⁻⁶ (Table 3), indicating gradual diffusion in the gastrointestinal tract.

**TABLE 3:** Pharmacokinetic properties of the ligands isocarapanaubine (AIN1) and 18‐β‐hydroxy‐3‐epi‐α‐yohimbine (AIN2) predicted by consensus absorption, distribution, metabolism, and excretion (ADME) testing between the PreADMET and ADMETlab platforms.

Compound	P_app_ Caco‐2 (in 10⁻⁶ cm/s)	P_app_ MDCK (in 10⁻⁶ cm/s)	PAMPA (P_eff_)	F%	BBB class. C_[brain]_/C_[blood]_	CL_int,u_ (mL/min/kg)
AIN1	2.869	0.304	—	97.01	0.085	6.18
AIN2	1.379	1.021	—	86.76	0.614	6.43

The prediction of ADME descriptors showed an estimated absorption fraction (F%) of 97.01% for AIN1 and 86.75% for the AIN2 derivative (Table 3), although the activity spectrum in the CNS is more favorable for AIN2 due to the alignment of physicochemical attributes (Table 2). This result corroborates with the predicted BBB permeability descriptors expressed in brain/blood concentration (C[brain]/C[blood]), with a predicted value of 0.6 for the AIN2 derivative [27] (Table 3).

### 2.7 Human Liver Microsome Stability Prediction

The prediction of phase I metabolic sites allows for the estimation of secondary metabolites formed in human liver microsomes (HLM), defining the metabolic profile of drug candidates and the potential toxic effects of these processes [28]. Biotransformations commonly associated with the CYP450 isoform system include oxidation, reduction, and dealkylation reactions at polar N‐ and O‐centers, as well as oxygenation reactions at aromatic centers, resulting in more polar metabolites for excretion [29]. However, some metabolites may be reactive and covalently bind to unwanted targets, such as proteins and DNA, causing liver damage [30]. These processes can also affect the oral bioavailability and hepatic clearance of these drug candidates [25].

In this analysis, it was observed that the heterocyclic ring containing a tertiary amine (R3N) is a CYP450‐dependent N‐dealkylation site in both ligands. The predictive ADME test showed that both can be substrates of major metabolizing isoforms in the human liver. However, the AIN1 derivative has two ─OCH_3_ groups attached to the aromatic ring that are susceptible to CYP450‐dependent O‐dealkylation biotransformation. On the other hand, the AIN2 ligand presents a greater number of phase II biotransformation sites, where the ─OH and ─COOCH_3_ groups are sites for glucuronidation and hydrolysis, respectively, potentially resulting in a low concentration of first‐pass secondary metabolites. These analyses suggest that AIN2 exhibits greater resistance to phase I metabolism, making the compound more metabolically stable compared to the AIN1 derivative.

## 3 Conclusions

Zebrafish assays demonstrated that the samples AIN1 and AIN2 were non‐toxic and exhibited anxiolytic behaviors at doses of 20 mg/kg for AIN1 and 12 mg/kg for AIN2, despite acting through different mechanisms. Molecular docking simulations suggested that AIN1 may act synergistically with other GABAAR receptor inhibitors, while AIN2 may modulate 5‐HT3AR receptors by interacting with common residues of the co‐crystallized inhibitor. Predictive DMPK tests revealed that the AIN2 derivative showed better alignment between permeability, metabolic stability, and safety for the CNS, although AIN1 also appears to be a viable candidate for anxiolytic action. Thus, this research contributes to the understanding that the indole alkaloids AIN1 and AIN2 should be further explored as potential tools in psychopharmacology.

## 4 Experimental

### 4.1 Drugs and Reagents

In this research, the following substances were used: FLX (Sandoz), DZP (Neo Química), DMSO (3%; Dynamic), GSTN, and FMZ (Sandoz). The indole alkaloid compounds used in the study were AIN1 and AIN2, the extraction and isolation from the *R. ligustrina* plant, as well as the determination of their chemical structure, were conducted by the research group and have already been described in the literature [31].

### 4.2 Zebrafish

Zebrafish aged between 90 and 120 days (0.4 ± 0.1 g), wild and of both sexes, were purchased from a store located in Fortaleza, CE. The animals were divided into groups of 50 individuals and acclimated in glass aquariums (30 × 15 × 20 cm) with dechlorinated water (ProtecPlus) and air pumps with submerged filters, maintained at 25°C, pH 7.0, and a light‐dark cycle of 10–14 h. The fish were fed with Spirulina food 24 h before the tests. At the end of the experiments, the fish were sacrificed by immersion in cold water (2–4°C) for 10 min until cessation of opercular movement. All activities involving the animals were approved by the Ethics Committee for the Use of Animals (CEUA) of the State University of Ceará (04983945/2021).

### 4.3 Acute Toxicity

The acute toxicity assessment was conducted in adult zebrafish according to the Organization for Economic Cooperation and Development (OECD) [32] standard testing method to determine the LD_50_ over 96 h. Each of the AIN1 and AIN2 samples was administered in doses of 4, 12, or 20 mg/kg via intraperitoneal (i.p.; 20 µL) injection, with each treatment dose group consisting of *n* = 6 fish. A negative control was DMSO (3%; 20 µL; i.p.) and a positive control was DZP (4 mg/kg; 20 µL; i.p.) were also used. Fish mortality was recorded every 24 h for each group to determine the LD_50_ [33].

### 4.4 Evaluation of Locomotor Activity

The open field test was conducted to investigate the influence of the studied samples on the locomotor activity of the animals. Each of the samples, AIN1 and AIN2, was administered at doses of 4, 12, or 20 mg/kg via intraperitoneal injection (i.p.; 20 µL), with each treatment dose group consisting of 6 fish. A negative control was DMSO (3%; 20 µL; i.p.) and a positive control was DZP (4 mg/kg; 20 µl; i.p.) were also used. After 30 min of treatment, the animals were placed in Petri dishes (10 × 15 cm), divided into four quadrants and filled with the same aquarium water, and the locomotor activity was assessed for 5 min by counting the number of LCs [20].

### 4.5 Anxiolytic Evaluation

The assay was conducted in a glass aquarium (30 × 15 × 20 cm) divided into a light and a dark area, filled with dechlorinated water to a height of 3 cm, simulating an environment different from a conventional aquarium and capable of inducing anxiety‐like behaviors. Each sample, AIN1 and AIN2, was administered in doses of 4, 12, or 20 mg/kg via intraperitoneal (i.p.; 20 µL) injection, with each treatment dose group consisting of six fish. A negative control was DMSO (3%; 20 µL; i.p.) and a positive control was DZP (4 mg/kg; 20 µL; i.p.) were also used. After 30 min, the animals were placed individually in the light area of the aquarium, and the anxiolytic effect was assessed based on the time spent in the illuminated area of the aquarium over 5 min [20]. At the end of the test, the optimal dose providing the desired activity without significant adverse effects for each sample was determined by comparing the anxiolytic test with the toxicity curve.

### 4.6 Evaluation of GABA_A_ Neuromodulation

The zebrafish (*n* = 6/group) was pre‐treated with FMZ (4 mg/kg; 20 µL; i.p.). After 15 min, the best anxiolytic dose of the sample, identified in the previous test, was administered. A group treated with DMSO (3%; 20 µL; i.p.) served as the negative control, and DZP (4 mg/kg; 20 µL; i.p.) served as the positive control and agonist of the benzodiazepine binding site on GABA_A_. After 30 min of treatment, the animals were subjected to the light/dark test, as described previously [34].

### 4.7 Evaluation of Serotonergic Neuromodulation

Groups of 6 fish were pretreated with the GSTN antagonist (5‐HT_3A_, 20 mg/kg, orally), and after 30 min, the fish received the optimal dose of the sample with anxiolytic effects. One group served as the negative control and received DMSO (3%; 20 µL; i.p.), while another group received FLX (0.05 mg/kg; i.p.), which was used as the positive control and agonist for the 5‐HT binding site. Finally, the groups were subjected to the light/dark test, as described in the “Anxiolytic Evaluation ” section [34].

### 4.8 Statistical Analysis

The results were expressed as mean values ± standard error of the mean for each group of 6 fish. After confirming the normality of the distribution and data homogeneity, differences between groups were analyzed using one‐way analysis of variance (ANOVA) for parametric data, followed by the Tukey test, and the Kruskal‐Wallis test was used for non‐parametric data. Two‐way ANOVA was employed in experiments with antagonists, followed by the Tukey test. All analyses were conducted using GraphPad Prism v. 10.3.1 software. The statistical significance level was set at 5% (*p < 0.05).

### 4.9 Molecular Docking Analysis

To theoretically analyze the anxiolytic mechanism of action, a series of independent molecular docking simulations were performed using the GABA_A_ (GABA_A_R) and serotonergic (5‐HT_3A_R) pathways. The “CryoEM structure of human full‐length alpha1beta3gamma2L GABA_A_R in complex with DZP (Valium), GABA, and megabody Mb38” and the “Cryo‐EM structure of the 5HT3A receptor in the presence of GSTN” were retrieved from the RCSB Protein Data Bank (https://www.rcsb.org/) under PDB ID codes 6HUP and 6NP0, respectively. These are classified as membrane receptors and present chemical structures resolved by electron microscopy at resolutions of 3.58 and 2.92 Å, respectively.

The protein preparation stage involved the removal of water residues and co‐crystallized small molecules, as well as the addition of polar hydrogens and Gasteiger charges, using the AutoDockToolsTM program (https://autodocksuite.scripps.edu/adt/). The grid box was adjusted to encompass the entire conformational space of the proteins, with dimensions of 94 × 92 × 126 Å along the axes x = 134.39, y = 135.69, and z = 133.776 for the GABA_A_R receptor, and dimensions of 86 × 90 × 126 Å along the axes x = 159.616, y = 159.619, and z = 144.899 for the 5‐HT_3A_R receptor. Finally, the AutoDockVinaTM code (https://vina.scripps.edu/) was configured to perform a cycle of 50 independent simulations of 20 docking poses each, using the Lamarckian Genetic Algorithm, where the selection criteria for the best pose included affinity energy parameters lower than ‐6.0 kcal/mol and a RMSD of less than 2.0 Å [35].

### 4.10 In Silico DMPK Analysis

To predict the properties of DMPK, analyses of the physicochemical attributes of the ligands AIN1 and AIN2 were conducted based on the CNS MPO developed by Pfizer, Inc., as shown in Equation (1):

(1) $$d=\sum_{i=1}^{N}w_{k}{\left(T\left(x^{0}\right)\right)}_{k}$$

w represents the weighting factor (ranging from 0 to 1) for each physicochemical attribute (k), associated with the satisfaction function of the statistical thresholds (T(x)). These thresholds include: intrinsic logP ≤ 3, lipophilicity at physiological pH (logD at pH 7.4) ≤ 2, MW ≤ 360 g/mol, TPSA between 40 and 90 Å^2^, number of HBDs ≤ 1, and basic pKa ≤ 8 (N = 6). The summation of these weightings results in a desirability score (d), ranging from 0 to 6, which indicates DMPK viability. The physicochemical properties were calculated using the academic licensed program MarvinSketch LTS version Neon.3, Chemaxon (https://chemaxon.com/marvin), and the results of the CNS MPO analyses were related to the predicted DMPK properties expressed in the Parallel Artificial Membrane Permeation Assay and ADME profile, utilizing the PreADMET (https://preadmet.qsarhub.com/adme/), ADMETlab (https://admetlab3.scbdd.com/), and ADMETboost—AI Drug Lab (https://ai‐druglab.smu.edu/admet) servers.

The prediction of stability in HLM was conducted through the prediction of the metabolism site of the ligands AIN1 and AIN2 using the XenoSite server (https://xenosite.org/) to identify phase I metabolism sites, dependent on cytochrome P450 (CYP450), and phase II, dependent on conjugation reactions.

## Author Contributions

**Nádia Aguiar Portela Pinheiro**: investigation, writing – review and editing. **Ivana Carneiro Romão and Amanda Maria Barros Alves**: supervision, formal analysis, software. **Sônia Maria Costa Siqueira and Andreia Ferreira de Castro Gomes**: writing the original draft and reviewing the manuscript. **Emmanuel Silva Marinho and Márcia Machado Marinho**: software and validation. **Jane Eire Silva Alencar de Menezes and Hélcio Silva dos Santos**: administration and project writing. **Herbert de Sousa Magalhães and Otília Deusdênia Loiola Pessoa**: extraction, isolation of samples, and characterization of samples.

## Conflicts of Interest

The authors declare no conflicts of interest.

## Data Availability

The data that support the findings of this study are available from the corresponding author upon reasonable request.
